# CD6-mediated inhibition of T cell activation via modulation of Ras

**DOI:** 10.1186/s12964-022-00998-x

**Published:** 2022-11-21

**Authors:** Sónia N. Henriques, Liliana Oliveira, Rita F. Santos, Alexandre M. Carmo

**Affiliations:** 1 i3S - Instituto de Investigação e Inovação em Saúde, Universidade do Porto, Rua Alfredo Allen, 208, 4200-135 Porto, Portugal; 2 IBMC - Instituto de Biologia Molecular e Celular, Porto, Portugal; 3 Programa Doutoral em Biologia Molecular e Celular (MCbiology), Instituto de Ciências Biomédicas Abel Salazar (ICBAS), Universidade do Porto, Porto, Portugal

**Keywords:** Cell surface molecules, Inhibitory receptors, Signaling transduction, Immunological synapse, GTPases, FRET

## Abstract

**Background:**

CD6 is one of many cell surface receptors known to regulate signal transduction upon T cell activation. However, whether CD6 mediates costimulatory or inhibitory signals is controversial. When T cells engage with antigen presenting cells (APCs), CD6 interacts with its ligand CD166 at the cell–cell interface while the cytosolic tail assembles a complex signalosome composed of adaptors and effector enzymes, that may either trigger activating signaling cascades, or instead modulate the intensity of signaling. Except for a few cytosolic adaptors that connect different components of the CD6 signalosome, very little is known about the mechanistic effects of the cytosolic effectors that bind CD6.

**Methods:**

Jurkat model T cells were transfected to express wild-type (WT) CD6, or a cytoplasmic truncation, signaling-disabled mutant, CD6Δcyt. The two resulting cell lines were directly activated by superantigen (sAg)-loaded Raji cells, used as APCs, to assess the net signaling function of CD6. The Jurkat cell lines were further adapted to express a FRET-based unimolecular HRas biosensor that reported the activity of this crucial GTPase at the immunological synapse.

**Results:**

We show that deletion of the cytosolic tail of CD6 enhances T-cell responses, indicating that CD6 restrains T-cell activation. One component of the CD6-associated inhibitory apparatus was found to be the GTPase activating protein of Ras (RasGAP), that we show to associate with CD6 in a phosphorylation-dependent manner. The FRET HRas biosensor that we developed was demonstrated to be functional and reporting the activation of the T cell lines. This allowed to determine that the presence of the cytosolic tail of CD6 results in the down-regulation of HRas activity at the immunological synapse, implicating this fundamental GTPase as one of the targets inhibited by CD6.

**Conclusions:**

This study provides the first description of a mechanistic sequence of events underlying the CD6-mediated inhibition of T-cell activation, involving the modulation of the MAPK pathway at several steps, starting with the coupling of RasGAP to the CD6 signalosome, the repression of the activity of Ras, and culminating in the reduction of ERK1/2 phosphorylation and of the expression of the T-cell activation markers CD69 and IL-2R α chain.

**Video abstract**

**Supplementary Information:**

The online version contains supplementary material available at 10.1186/s12964-022-00998-x.

## Introduction

CD6 is a type I transmembrane glycoprotein expressed mainly by peripheral T lymphocytes, medullary thymocytes and B1a lymphocytes [1–3]. It remains controversial whether CD6 is a co-stimulatory or inhibitory receptor as, depending on the particular cellular [4–8] or animal [9–11] model, CD6 has been reported to either activate or repress T-cell responses. It may well be that, contingent on the cell subset where it is expressed and the context or phase of the immunological response, CD6 would behave as a dual-function molecule [12].

Upon T cell receptor (TCR) triggering, tyrosine residues of the cytosolic tail of CD6 are rapidly phosphorylated and become putative docking sites for specific SH2 domain-containing enzymes and adaptors [13]. These reactions allow CD6 to assemble a large signalosome that may include both positive as well as negative [14–18] regulators of T-cell activation, and also intracellular adaptors such as SLP-76 and TSAD [19, 20]. Although the combination of all these mediators has the potential to balance signaling strength and fine-tune T cell activation [21], it has been shown that in the absence of the CD6 cytosolic tail, T cells or T cell lines respond more vigorously to stimulation [8] suggesting that, mechanistically, the overall effects of CD6 correspond to those of an inhibitory membrane scaffold protein.

CD6 shares with the related T-cell antigen CD5 many genetic, structural, and functional characteristics [21]. Long established as an inhibitory receptor [22], CD5 was suggested to constrain T-cell signaling due to its association with the SH2 domain-containing activation-repressive effectors E3 ubiquitin-protein ligase CBL, Ras GTPase-activating protein 1 (RasGAP), and protein-tyrosine phosphatase SHP-1 [23–25]. Recently, also CD6 was shown to be coupled by SHP-1 upon direct extracellular ligation with its conventional ligand, CD166 [17]. However, the contribution of these effectors towards CD5- or CD6-mediated signaling has only been inferred and not formally demonstrated. Indeed, the actual participation of SHP-1 in CD5 function has recently been challenged, since no differences in CD5-based signaling between thymocytes from SHP-1‑deficient mice and thymocytes from SHP-1-positive control mice were observed [26].

A key event in TCR-induced signaling is the activation of the family of small GTPases collectively known as Ras proteins [27, 28]. The most important Ras members are encoded by the genes *HRAS*, *NRAS* and *KRAS* [29, 30], and the fundamental biological role of this family of GTPases is highlighted by the fact that mutations in RAS genes are found in over 25% of all human cancers [31, 32]. Ras proteins share 90% identity in the first 165 aa, which are followed by a hypervariable region (HVR) of 20 aa (19 aa in KRas2B, the predominant of two alternative-splicing generated isoforms of the *KRAS* gene) and end with a conserved C-terminal CAAX box (in which C = cysteine, A = aliphatic, and X = any but commonly serine or methionine). Although the different Ras proteins and isoforms display subtle differences in their subcellular localization [33] and traffic between different endomembrane compartments [34, 35], they can all respond to mAb-mediated TCR triggering, albeit with different sensitivities, and seem capable of colocalizing with the TCR/CD3 complex at the plasma membrane (PM) of T cells or T cell lines [36, 37]. It remains disputed, however, whether Ras proteins are preferentially activated at the PM or on the Golgi apparatus [36–39].

Ras proteins cycle between GTP-bound active and GDP-bound inactive forms, and are activated upon TCR triggering through the coincident action of specific guanine nucleotide exchange factors (GEFs), such as SOS-1 and RasGRP1 [40, 41], and the repression or exclusion of GTPase-activating proteins (GAPs), such as RasGAP and neurofibromin which accelerate the hydrolysis of Ras-bound GTP [27, 42]. The best characterized Ras effector is the serine/threonine-protein kinase Raf-1 [43, 44], but activation of Ras feeds into multiple signaling pathways that promote the activation of transcription factors such as AP-1 and NFAT [45–47], and the upregulation of genes encoding critical factors for T-cell proliferation and effector functions, including the T cell-growth factor IL-2, the α chain (CD25) of the high-affinity IL-2 receptor, and the early activation marker CD69 [48–51].

We investigated the interaction between CD6 and putative downstream signaling effectors and show in this study that CD6 interacts at the cytosolic level with RasGAP in a phosphorylation-dependent manner and, importantly, that deletion of the cytosolic tail of CD6 results in an increase in the GTPase activity of HRas at the immunological synapse (IS), as assessed using a novel HRas biosensor based on Förster resonance energy transfer (FRET). This study thus constitutes the first clear evidence for a molecular mechanism underlying the inhibitory function of CD6, highlighting the critical role of this receptor in modulating the strength of signals originating at the immediate steps after TCR triggering, and in regulating T cell activation overall.

## Material and methods

### Cell lines and cell culture

Jurkat E6.1 and Raji cells were cultured in RPMI-1640 (Cytiva) supplemented with 10% (v/v) fetal bovine serum, 1% (v/v) penicillin–streptomycin and 1% (v/v) sodium pyruvate (all from Thermo Fisher Scientific). HEK293T cells were cultured in DMEM (Cytiva), supplemented as RPMI. Cell cultures were kept in a controlled incubator (BINDER) at 37 °C and 5% CO_2_.

### CD6 constructs and expression in E6.1 cells

CD6-encoding sequences were amplified by PCR from pEGFP-N1/CD6FL [52] and corrected to coincide with the GenBank accession no. U66142.1 by point mutating the appropriate single residues. The sequence encoding the cytosolic deletion mutant CD6Δcyt includes a stop codon to substitute for K429, and still maintains 5 aa of the cytosolic tail-sequence for proper membrane attachment and stability. cDNA sequences were cloned via *Bam*HI/*Mlu*I and *Not*I restriction sites into the lentiviral expression vector pHR, which was then transfected into HEK293T cells along with p8.91 and pMDG for lentiviral production, as described elsewhere [53]. After 72 h, the medium was collected, filtered and used to stably transduce E6.1 cells. All transfected cell lines were checked for CD6 expression level homogeneity by flow cytometry and, when needed, cell sorting was performed based on CD6 and CD3 expression.

For flow cytometry analyses, 0.5 × 10^6^ parental or transfected E6.1 cells were collected and washed twice in cold flow cytometry buffer [0.2% (w/v) BSA (NZYTech), 0.1% (v/v) sodium azide (Merck) in PBS (Thermo Fisher Scientific)]. Cells were then incubated with FITC-conjugated CD6 mAb (BL-CD6, BioLegend) for 30 min at 4 °C. After washes, cells were filtered with 100 μm nylon filters (Corning Life Sciences) into FACS tubes. Acquisition was performed on a BD Accuri™ C6 Flow Cytometer (BD Biosciences) and analyzed using the FlowJo™ v10 Software (BD Biosciences).

### Activation of E6.1 cells

Superantigen-mediated T cell activation was performed as previously described [54], using 5 × 10^4^ Raji cells pre-loaded with 1 μg/ml staphylococcal enterotoxin E (SEE, Toxin Technology) co-cultured with 5 × 10^4^ CD6 wild-type (WT)- or CD6Δcyt-expressing E6.1 cells, for 24 h at 37 °C. In parallel, E6.1 cells were also co-cultured with unloaded Raji cells, or RPMI alone. Cells were then stained with APC-conjugated CD69 (FN-50) or CD25 (BC96) mAbs (all from BioLegend), and cell activation was measured by flow cytometry. Raji cells were gated out using PeCy7-conjugated CD19 mAb (HIB19, Thermo Fisher Scientific).

E6.1 cell activation using pervanadate was performed as described [55], using 50 × 10^6^ cells. Cells were activated for 5 min at 37 °C, pelleted for 30 s, the supernatants discarded, and then cells were lysed for 30 min on ice in 500 μl of lysis buffer [1% (v/v) Triton X-100, 10 mM Tris⋅Cl pH 7.4 (NZYTech), 150 mM NaCl (NZYTech), 1 mM EDTA (Merck), 1 mM PMSF (Merck), 1 mM orthovanadate (Sigma)]. Lysates were centrifuged for 10 min at 11,500 × *g* at 4 °C and the supernatants were recovered for further analysis. 50 μl of lysate were mixed with 50 μl 2 × Laemmli buffer (Bio-Rad), with or without β-mercaptoethanol, and denatured for 5 min at 95 °C, to be run in SDS-PAGE. The remaining 450 μl of lysate were pre-cleared with 50 μl of Protein A Sepharose® CL-4B reconstituted beads (Cytiva) for 45 min at 4 °C by end-over-end rotation. Beads were pulled down by centrifugation at 500 × *g* for 1 min, the lysates recovered and then used for immunoprecipitation with 100 μl of Protein A Sepharose beads pre-packed with 1 μg of CD6 mAb (MEM98, EXBIO). Immunoprecipitates were washed three times with 500 μl lysis buffer. Beads were then resuspended in 50 μl of 2 × Laemmli buffer and denatured for 5 min at 95 °C for use in SDS-PAGE.

For E6.1 cell activation using mAbs, 5 × 10^6^ E6.1 cells were harvested, washed with cold PBS, and kept on ice until activation. Cells were simultaneously activated for 5 min at 37 °C with CD3 (UCHT1) and CD28 (CD28.2) mAbs (all from BioLegend), at 3 and 5 μg/ml, respectively. Cells and lysates were then processed as above.

### SDS-PAGE and western blotting

Protein samples were separated in polyacrylamide gels (7 to 12%, depending on the target protein) and transferred to nitrocellulose membranes using the Trans-Blot Turbo Transfer System (Bio-Rad). Western blotting (WB) was performed as described previously [56]. Primary antibodies used were anti-CD6 mAb (MEM98, EXBIO), anti-RasGAP mAb (B4F8, Santa Cruz Biotechnology), anti-p21 Ras mAb (Cytoskeleton, Inc), anti-α-tubulin mAb (B-5-1-2, Merck), anti-ERK 2 rabbit polyclonal (C14, Santa Cruz Biotechnology), and Phospho-p44/42 MAPK rabbit polyclonal (Cell Signaling Technology); secondary antibodies used were HRP-conjugated goat anti-mouse IgG (BioLegend), rat anti-mouse IgG (Abcam) for detection of CD6 in MEM98 immunoprecipitates, and mouse anti-rabbit IgG (Santa Cruz Biotechnology). Membranes were developed with Amersham ECL Select/Prime Western Blotting Detection Reagent (Cytiva). Data were acquired using the ChemiDoc XRS+ System (Bio-Rad) and analyzed by Fiji [57].

### Construction of the unimolecular HRas/Raf-1 FRET biosensor MRS2

MRS2 was generated by assembling elements from GTPase FRET biosensors previously created and validated. Sequences for HRas (aa 1–172) and Raf-1 (aa 51–131) were retrieved from the described Raichu-Ras sensor (GenBank accession no. AB046925) [58]. The sequence for Raf-1 was modified in three nucleotides to match the GenBank accession no NM_002880.4. The split sequences from HRas [N-terminal, aa 1–172; C-terminal (CT), aa 173–189] included in the construct are according with the GenBank accession no NM_001130442.3. Sequences for Clover, mRuby2, linker 1 (L1), L3 and L4 were retrieved from the pCAGGS-Raichu-RhoA-CR plasmid [59]. pCAGGS-Raichu-RhoA-CR was a gift from Michael Lin (Addgene plasmid #40258; http://n2t.net/addgene:40258; RRID:Addgene_40258). L2 was amplified by PCR from an optimized version of the Raichu-RhoA probe [60], kindly given by João Relvas (i3S, Porto).

For assembly, two sections of MRS2 were synthesized: from Clover to HRas, and from Raf-1 to HRas-CT. Both fragments and the L2 were assembled using the NEBuilder® HiFi DNA Assembly Master Mix (New England Biolabs Inc.). MRS2-S17N was obtained via site-directed mutagenesis of MRS2 by PCR. Constructs were cloned into the pHR vector via the restriction site *Mlu*I, as described [53]. All plasmids were verified by sequencing.

### Cellular distribution of MRS2

1 × 10^5^ MRS2- and MRS2-S17N-expressing E6.1 cells were plated on glass coverslips previously coated with 0.01% poly-L-lysine (Merck) in PBS, for 30 min at 37 °C. After adhesion, the medium was removed and cells were fixed with paraformaldehyde 2% (Electron Microscopy Sciences) in PBS for 10 min, and thoroughly washed. Cells were then permeabilized with 0.1% Triton X-100 in PBS for 10 min and washed. Nuclei were stained with DAPI. Samples were washed and mounted on coverslips using Vectashield mounting medium (Vector Laboratories). Images were acquired in a Zeiss Axio Imager Z1 microscope (Carl Zeiss, Germany) equipped with an Axiocam MR ver3.0 camera (Carl Zeiss) and a Plan-Apochromat 63x/1.40 Oil DIC objective, and controlled through the Axiovision 4.9 software (Carl Zeiss, Germany).

### Live cell imaging of immunological synapses

5 × 10^4^ E6.1-MRS2 and E6.1-MRS2-S17N cells were washed twice with RPMI without phenol red (Cytiva), resuspended in the same medium, and plated on poly-L-lysine-coated µ-slides (ibidi) for 30 min at 37 °C. Meanwhile, 1 × 10^5^ Raji cells were incubated with SEE (1 μg/ml) in RPMI for 45 min at 37 °C, and posteriorly washed. Slides containing the biosensor-expressing E6.1 cells were placed under the microscope for imaging and initially filmed for 5 min, at which point acquisition was paused and sAg-loaded Raji cells were carefully added to the plate. Acquisition was resumed and proceeded for an additional 30 min.

Sequential frames were acquired every minute using an inverted motorized Nikon Ti widefield microscope (Nikon) equipped with an Andor iXon888 EMCCD camera (Andor), a PL APO LAMBDA 60x/1,4 Oil DIC objective, a Lumencor SpectraX light source, and controlled through the NIS-Elements AR software (version 5.02). All acquisitions were performed in a controlled environment at 37 °C and 5% CO_2_ using an Okolab microscope incubator system (Okolab). To image the Clover fluorophore emission, the lumencor Spectra X cyan filter 470/24 was used for excitation with a GFP/DsRed—bs 493/574, and a 514/30 emission filter. For mRuby2, the lumencor Spectra X green filter 550/15 was used for excitation with a GFP/DsRed—bs 493/574, and a 617/73 emission filter. For the FRET channel, the lumencor Spectra X Cyan filter 470/24 was used for excitation, a GFP/DsRed—bs 493/574, and a 617/73 emission filter. All LEDs were used at 10%. All images from fluorescence channels were acquired with no binning and 100 ms exposure time. Additionally, a brightfield image was acquired with the lamp at 6.5 V and an exposure time of 10 ms.

## Results

### CD6 modulates CD69 and CD25 upregulation following T cell activation

**Fig. 1:**
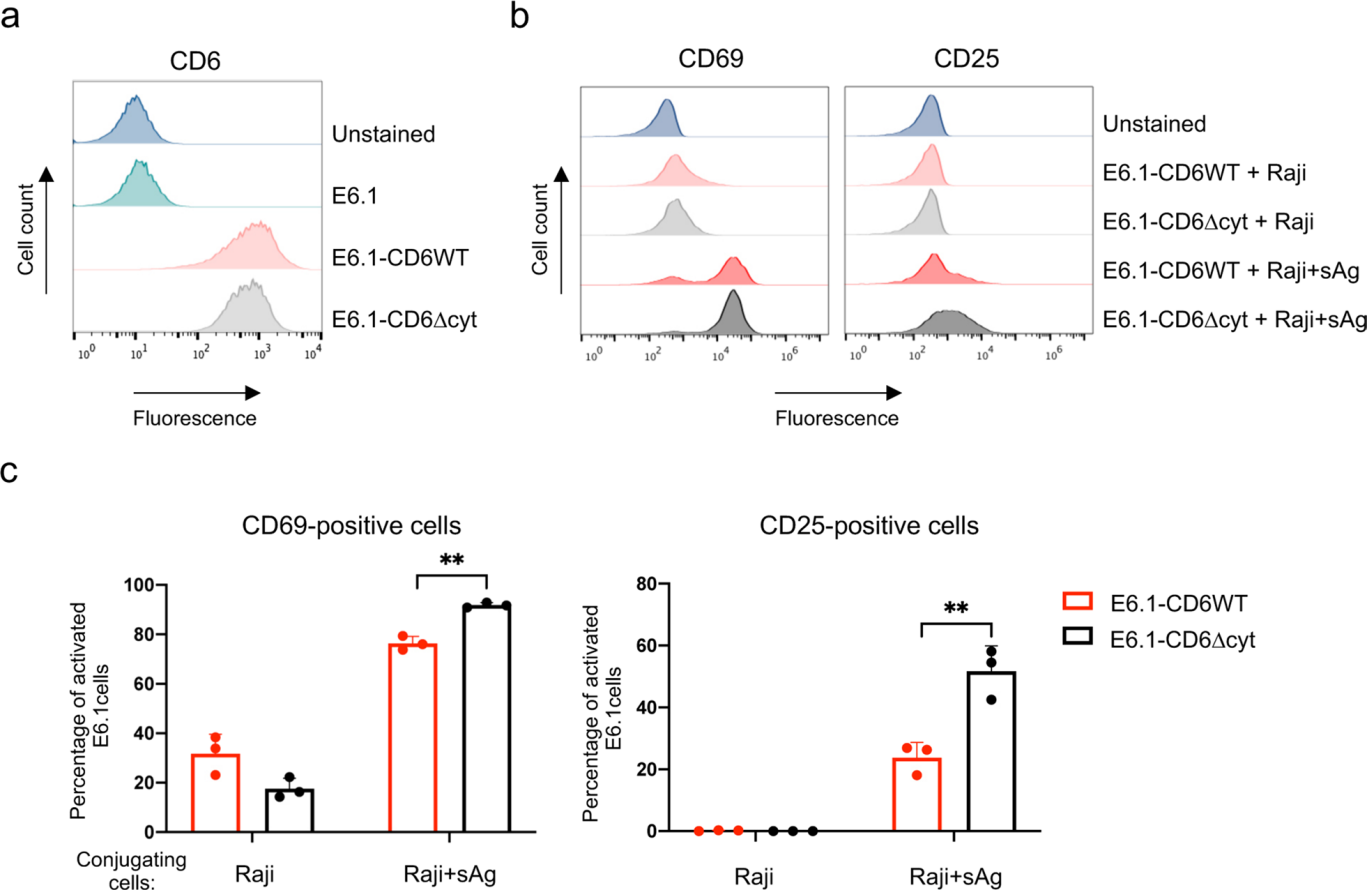
CD6 inhibits CD69 and CD25 upregulation following T cell activation. **a** E6.1 cells were stably transduced with vectors encoding CD6WT or CD6∆cyt. Cells were stained with fluorochrome-conjugated CD6 mAbs, and protein expression was assessed by flow cytometry. Histograms show the expression of the corresponding CD6 forms in each cell line. **b** E6.1-CD6WT and E6.1-CD6Δcyt cells were cultured for 24 h in the presence of unloaded or sAg-loaded Raji cells, and expression of CD69 and CD25 was then assessed by flow cytometry. Shown are representative histograms of CD69 and CD25 expression of one of three independent experiments. **c** Percentage of CD69-positive (left panels) and CD25-positive (right panels) E6.1-CD6WT or E6.1-CD6ΔDcyt cells cultured with Raji cells without or with sAg. Each dot represents one independent experiment. Bars represent mean and SD for each condition, that were compared using a paired *t* test. **, *p* < 0.01

We set-up an in vitro cellular system in which T-cell activation could be measured as the upregulation of the T-cell responsive antigens CD69 and CD25 in Jurkat E6.1 cells interacting with superantigen (sAg)-loaded Raji cells, here used as antigen presenting cells (APCs). Jurkat cells express only residual levels of endogenous CD6 (Fig. 1a), so they constitute an excellent experimental model in which to express CD6 variants and mutants to assess the function of the different components of the molecule. Expression plasmids encoding the wild-type (WT) CD6 molecule and a cytosolic-truncation mutant (CD6Δcyt) were transduced into E6.1 cells, which then expressed these CD6 forms at equivalent levels (Fig. 1a), and each of these cell lines was subjected to interaction with Raji cells previously incubated, or not, with sAg. As seen in Fig. 1b, c and Additional file 1: Fig. S1, E6.1-CD6WT and E6.1-CD6Δcyt cells were strongly activated only in the presence of Raji cells pre-incubated with sAg. Moreover, E6.1-CD6Δcyt cells were more strongly activated than E6.1-CD6WT cells, as assessed by the increased fraction of cells expressing CD69 and CD25. This indicates that the cytosolic tail of CD6 is responsible for reducing the strength of activation, confirming our previous findings [8].

### CD6 interacts with RasGAP in a phosphorylation-dependent manner

**Fig. 2:**
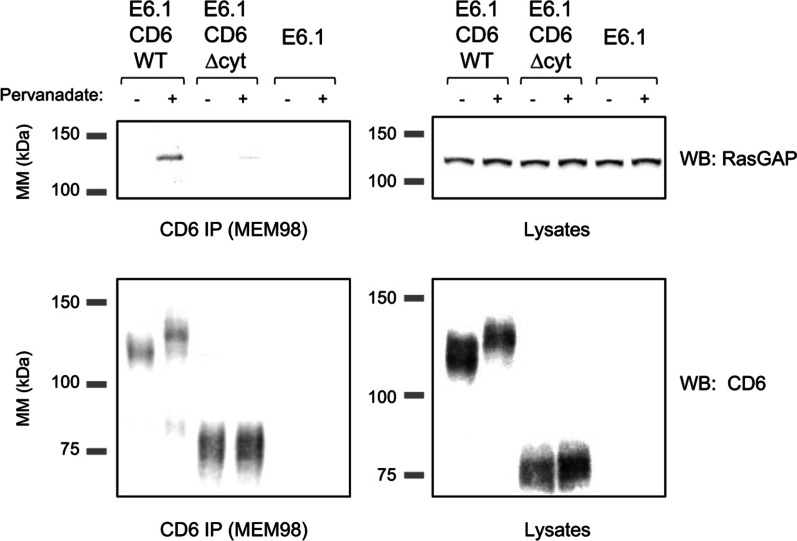
RasGAP associates with the cytoplasmic tail of CD6 depending on tyrosine phosphorylation. E6.1-CD6WT, E6.1-CD6∆cyt, as well as untransfected E6.1 cells were incubated with pervanadate for 5 min at 37 °C, lysed in Triton X-100 lysis buffer, and CD6 was immunoprecipitated. RasGAP was detected by WB in CD6WT but not in CD6∆cyt immunoprecipitates in activated cells (left panels). WB of cell lysates (right panels) confirms the presence of the indicated molecules at equivalent levels. Images are of one of two independent experiments. MM, molecular mass; IP, immunoprecipitation; WB, western blotting

Among other potential effector candidates that we tested in preliminary assays, we found that RasGAP interacted with CD6 in a tyrosine-phosphorylation dependent manner (Fig. 2). E6.1-CD6WT cells were activated with pervanadate, a potent and selective inhibitor of phosphotyrosyl-protein phosphatases which allows for the extensive tyrosine phosphorylation of intracellular proteins and is particularly effective in inducing overt T-cell activation [55, 61]. RasGAP was co-precipitated with CD6WT only upon cell activation (Fig. 2, top left). We further established that the interaction between CD6 and RasGAP is mediated by the cytosolic tail of CD6 since in E6.1 cells expressing CD6∆cyt, RasGAP was not co-precipitated with the mutant CD6 molecule before or following activation.

### Design, expression and activity of the FRET sensor MRS2, which reports on HRas activation upon T-cell stimulation by sAg-loaded APCs

**Fig. 3:**
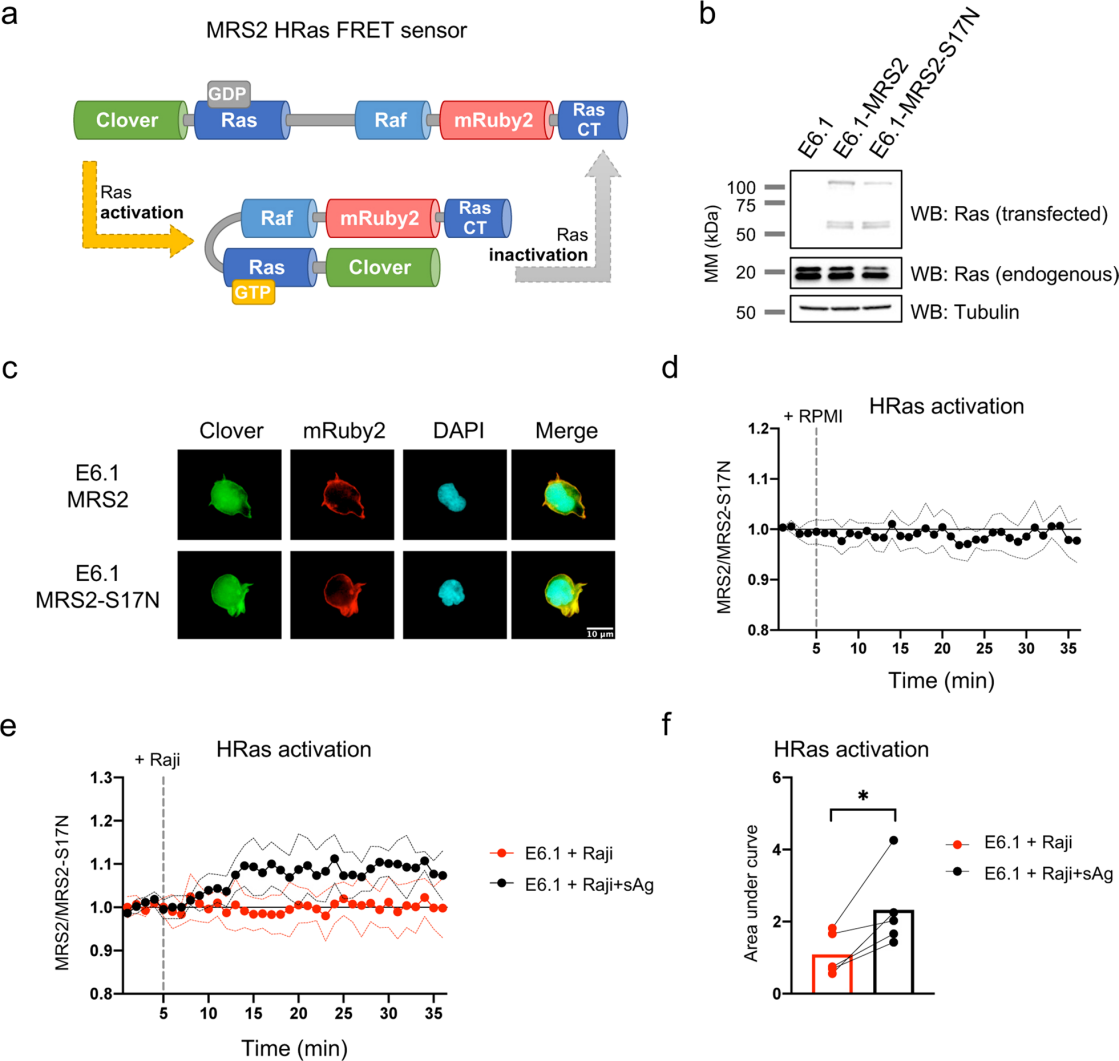
The FRET biosensor MRS2 reports on HRas activation. **a** Schematic representation of the MRS2 fusion protein and its conformation when HRas is active or inactive. **b** MRS2- and MRS2-S17N-encoding plasmids were stably transduced into E6.1 cells, and the expression of the fusion proteins was assessed by WB, displaying a size of ~ 120 kDa. Protein species of 58 and 60 kDa presumably represent proteolytic cleavage by-products of the full probe. **c** Distribution of MRS2 and MRS2-S17N in E6.1 cells assessed by fluorescence microscopy. **d** MRS2- and MRS2-S17N-expressing E6.1 cells were filmed for 5 min at 37 °C (1 min frames), medium was then added, and cells were filmed for a further 30 min (see Additional file 2: Movie S1). FRET/Clover ratio was assessed for each time-point, and the ratio from MRS2-expressing E6.1 cells was normalized to that of MRS2-S17N-expressing cells. **e** E6.1 cells expressing MRS2 and MRS2-S17N formed conjugates with Raji cells, unloaded or pre-loaded with sAg (see Additional file 3: Movie S2). Raji cells were added at the 5 min mark. FRET/Clover ratios from MRS2-expressing E6.1 cells were divided by those of MRS2-S17N-expressing E6.1 cells for each condition, and normalized for the average of the first three time points to establish the basal activity before the addition of Raji cells. **d**, **e** Dots and lines represent mean ± SD of 5 independent experiments. In each experiment, 10 individual cells (in **d**) or 9–10 cell pairs (in **e**) were assessed for both MRS2 and MRS2-S17N biosensors. **f** Area under the curve of graph in (**e**), with the baseline starting at 1. Each dot represents one of 5 independent experiments, and lines represent paired experiments. 9–10 cells were assessed in all conditions of each individual experiment. *, *p* < 0.05, paired *t* test

Having established that CD6 recruits the signaling-inhibitory mediator RasGAP upon T-cell activation, and created a cellular system wherein CD6 clearly suppresses T-cell signaling, we next assessed whether Ras could be influenced by CD6 function. We thus created and optimized a biosensor to track Ras and measure changes in its activity in real time using FRET microscopy. The unimolecular FRET reporter we developed, MRS2, was modified from the original “Ras and interacting protein chimeric unit” (Raichu) probes [58] by: (1) replacing the cyan and yellow fluorescent proteins (FP) for the much brighter Clover (green) and mRuby2 (red) FPs [59], which confer greater photostability to FRET reporters, have a wider FRET dynamic range and higher sensitivity to detect ratio changes, and may be less phototoxic to live imaged cells [59, 62, 63]; (2) inserting, based on the studies of Komatsu et al*.* [64], an extended flexible linker between the sequence of HRas and that of the Ras-binding domain (RBD) of Raf-1, to reduce the basal FRET signal and thereby increase the gain of FRET biosensors; and (3) adding the C-terminal sequence of HRas to target the reporter to the proper subcellular compartments. MRS2 thus comprises, in the following order, the FP Clover, HRas amino acids M1-N172, a 129 aa-long linker, the RBD of Raf-1, the FP mRuby2, and the C-terminal aa P173-S189 of HRas (Fig. 3a). To control for variations in MRS2 output arising from experimental artifacts, namely a predicted accumulation of the probe at the IS resulting in increased FRET that did not result from the activation of Ras (*i.e.*, bystander FRET), we generated a second, inactive, sensor (MRS2-S17N), wherein a point mutation of Ser17 of HRas precludes its activation [65].

E6.1 cells were stably transduced with MRS2- or MRS2-S17N-expressing plasmids and the expression of the full length biosensors was confirmed by WB (Fig. 3b). In addition to the full-length proteins, having a molecular weight (MW) of ~ 120 kDa, other species that were approximately half the size of the probe (58 and 60 kDa) could be detected in the blots, likely resulting from proteolytic cleavage. Transduced cells were imaged by fluorescence microscopy to confirm the proper cellular distribution of the probes, and whereas mRuby2 could only be detected at the plasma membrane, Clover was distributed between the membrane and the cytosol (Fig. 3c). This suggested that a fraction of the proteins can indeed be cleaved and while the N-terminal half may diffuse within the cytoplasm, the C-terminal hemi-probe, because it contains the membrane-associating C-terminal domain of HRas, remains localized to the membrane.

The probes that remain intact should nonetheless be bound to the membrane. To confirm that the integral probe is functional and able to report on the activity of HRas over time, we plated the transduced cells on poly-L-lysine-coated coverslips and filmed them for 35 min under a fluorescence microscope, in the absence of any type of stimulation. The activity of the biosensors was only detected at the cell surface (Additional file 2: Movie S1) and is presented as the ratio between FRET and Clover emissions, quantified at the single cell-level. This ratio at steady-state (basal level) increased slightly over time but remained stably and consistently higher than that of MRS2-S17N (Additional file 1: Fig. S2a). Normalization of data from MRS2 cells to that of the control MRS2-S17N cells demonstrated that the sensor remained stable over the course of the experiment in the unstimulated cells (Fig. 3d).

We next assessed whether MRS2 is able to report on HRas activation and localization during cell-mediated antigen presentation. Biosensor-transduced E6.1 cells were plated and filmed as they interacted with either unloaded or with superantigen (sAg)-loaded Raji cells (Additional file 3: Movie S2). Whereas in the occasional contacts between E6.1 and unloaded Raji cells there was no clear accumulation of either the MRS2 or MRS2-S17N biosensors at the cellular interfaces, in the presence of sAg, Raji cells were stably adhered to by E6.1 cells, and both of the probes accumulated at the IS during the period of the interactions. The FRET signals reported by MRS2 (Additional file 1: Fig. S2b) were normalized to the corresponding data from MRS2-S17N cells, revealing that while E6.1 cells interacting with unprimed Raji cells did not exhibit HRas activation, the formation of a synapse between E6.1 cells and sAg-loaded Raji resulted in a sustained elevation of HRas activity (Fig. 3e, f). Therefore, the MRS2 FRET sensor is capable of reporting on the activation and localization of HRas upon T-cell recognition of APC-presented antigen.

### The cytosolic tail of CD6 constrains HRas activation and downstream ERK1/2 phosphorylation

**Fig. 4:**
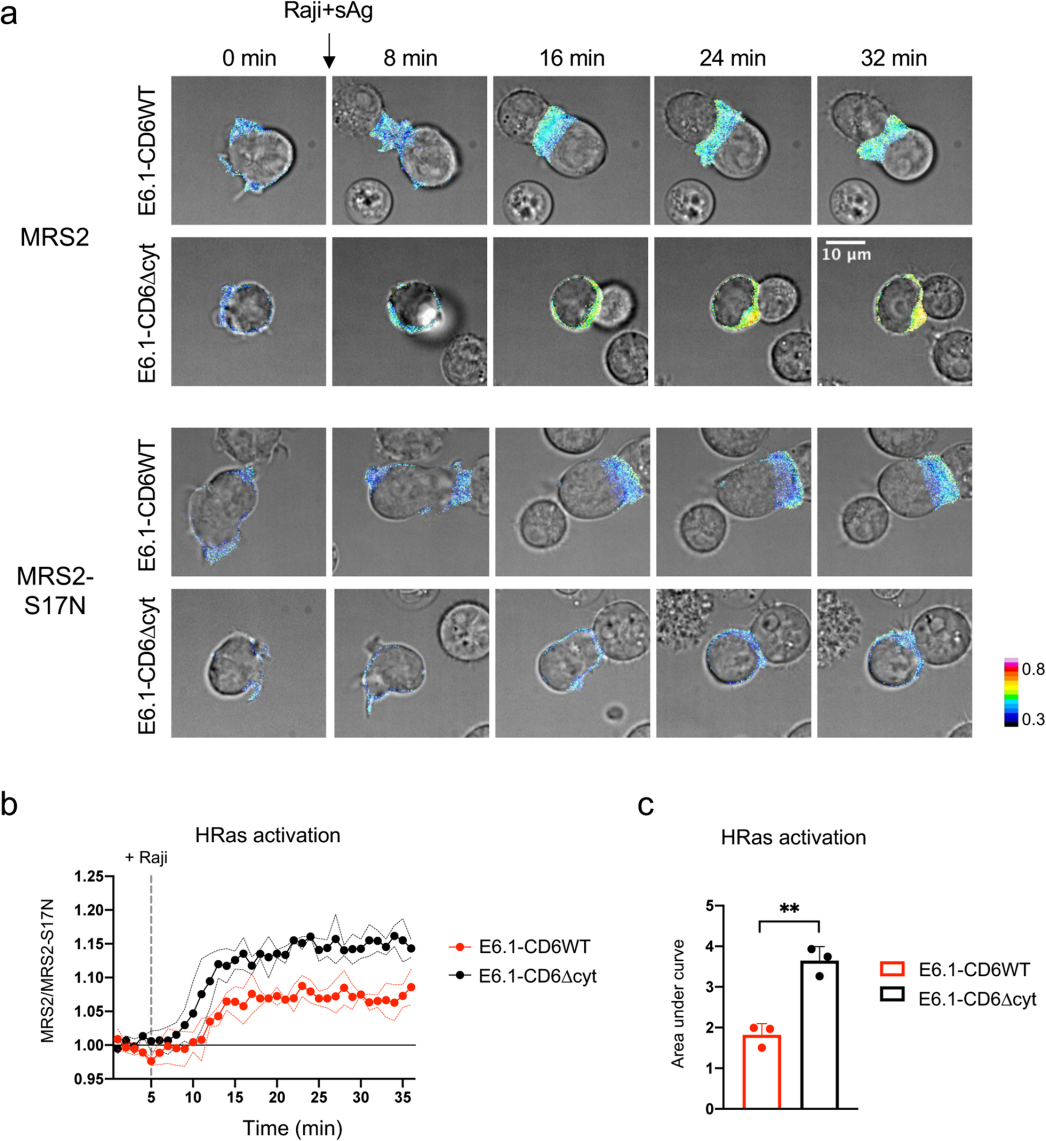
The cytosolic tail of CD6 constrains HRas activation in E6.1 cells upon APC-mediated activation. **a** Representative images of E6.1-CD6WT and E6.1-CD6∆cyt cells, expressing either MRS2 or MRS2-S17N, during the formation of conjugates with sAg-loaded Raji cells (full movies at Additional file 4: Movie S3). FRET/Clover ratio is presented as a thermal range (scale in bottom right). **b** FRET/Clover ratios from MRS2-expressing cells (see Additional file 1: Fig. S2c) were divided by those of MRS2-S17N-bearing cells and normalized to the average of the first three points before the addition of Raji cells. Dots and lines represent mean ± SD of 3 independent experiments. **c** Area under the curve of graph in B, where baseline = 1. Bars represent mean and SD, and each dot represents an independent experiment. 9–10 cells were assessed in all conditions of each individual experiment. Comparisons were performed using a paired *t* test. **, *p* < 0.01

The potential regulation of Ras activation by CD6 was then investigated in our cellular system, with E6.1 cells expressing either the MRS2 or the MRS2-S17N biosensors being further transduced with plasmids encoding CD6WT or the cytosolic truncation mutant CD6∆cyt. E6.1-CD6WT-MRS2 and E6.1-CD6∆cyt-MRS2 cells were placed in culture with Raji cells pre-incubated with sAg and imaged during the formation of synaptic contacts for 30 min (Fig. 4a and Additional file 4: Movie S3). As cellular contacts were established and T-cell activation progressed, the MRS2 biosensor concentrated at the synapses for the duration of the assays. Strikingly, the level of HRas activation in E6.1-CD6∆cyt-MRS2 cells was higher than that of the corresponding CD6WT-expressing cells. By normalizing the FRET/Clover ratios by those of the corresponding CD6WT or CD6∆cyt cells expressing the silent MRS2-S17N biosensor (Additional file 1: Fig. S2c), we could calculate that CD6WT-expressing cells exhibited 50% lower activation of HRas upon IS assembly than CD6∆cyt-expressing cells over the course of the 30 min assay (Fig. 4b, c).

**Fig. 5:**
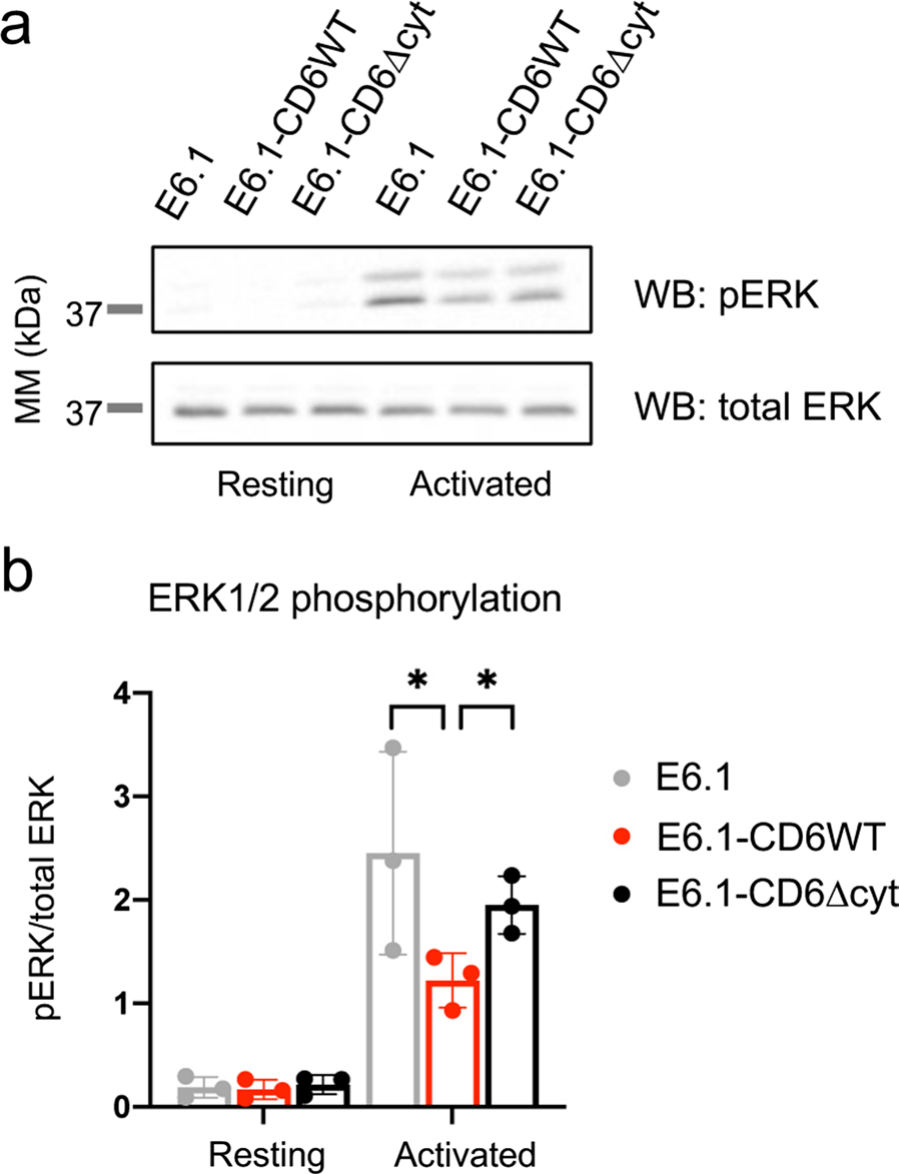
CD6 downmodulates ERK1/2 phosphorylation in activated cells. **a** Parental E6.1 cells, E6.1-CD6WT and E6.1-CD6∆cyt cells were left undisturbed or were activated with CD3 (3 μg/ml) and CD28 (5 μg/ml) mAbs for 5 min at 37 °C, and lysed. Total and phosphorylated ERK1/2 levels were detected in lysates by WB, and quantified using Fiji. **b** Quantification of ERK1/2 phosphorylation from three independent experiments. Bars represent mean and SD. The probability that the phosphorylation of ERK was similar between E6.1-CD6WT and parental E6.1 or E6.1-CD6∆cyt cells was assessed using a paired *t* test. *, *p* < 0.05

We next assessed in E6.1-CD6WT, E6.1-CD6∆cyt and also parental E6.1 cells the levels of phosphorylation of the extracellular signal-related kinases (ERK)-1/2, downstream of Ras in the MAPK pathway. Cells were activated with anti-CD3 and anti-CD28 mAbs, and phosphorylated ERK1/2 was detected by WB (Fig. 5a). As can be seen, the ratio of phosphorylated to total ERK1/2 was significantly lower in E6.1-CD6WT cells than in E6.1 or E6.1-CD6∆cyt cells after activation (Fig. 5b). This indicates that CD6, and more particularly its cytosolic tail, is responsible for restraining the whole of the MAPK pathway, consistent with the observed effects of the repression of HRas at the initiation of the signaling cascade.

## Discussion

For the past decade the role of CD6 on T-cell activation has been vigorously debated. Previous reports indicated that CD6 might function as a co-stimulatory protein that amplifies TCR-mediated activation [5–7, 66, 67]. Most of the studies utilized CD6 antibodies to address CD6 function but, as now seems clear, the effects of antibodies are not always clear cut and may produce experimental artifacts. Firstly, CD6 antibodies were routinely used in multicellular assays in order to block the CD6-CD166 interaction, whereas the antibodies were subsequently found to be non-blockers (reviewed in [68]). Secondly, the use of antibodies to cross-link CD6 may in fact result in the extensive aggregation of the CD6 cytosolic tail-associated signalosome, overriding any subtle regulatory effects that physiologically engaged CD6 might otherwise have.

The functional role of CD6 now has translational implications. In recent years, CD6 has emerged as a potential target for therapy in several autoimmune indications such as multiple sclerosis, Sjögren's syndrome and asthma [69–71], and the humanized anti-CD6 monoclonal antibody Itolizumab is already in clinical use to treat chronic plaque psoriasis, rheumatoid arthritis and COVID-19 [72–74]. It had been conjectured that Itolizumab modulates T-cell activation by disrupting the interaction between CD6 and its APC-expressed ligand CD166 [17]. However, using functional assays and biophysical approaches we have shown that Itolizumab does not physically block the CD6-CD166 interaction [54]. It therefore remains undetermined whether the antibody represses T-cell activation by interfering with the physiological function of a “co-stimulatory” CD6 molecule or by eliciting the “inhibitory” potential of CD6.

Notwithstanding the beneficial effects of CD6 mAbs and the investigation of the mechanisms underlying CD6-targeted therapy in the clinic, there is still much to be learned from basic, molecular studies of CD6. Using an experimental system constituted by APCs presenting sAg to primary T-cells or T-cell lines in which we manipulated the expression of CD6, we previously suggested that CD6 constrains T-cell activation on the basis that it decreases calcium signaling in cells expressing full-length CD6, comparing with cells expressing a CD6 cytosolic deletion mutant [8]. In the present study using FRET microscopy in Jurkat T cells, we now show that the cytosolic tail of CD6 is responsible for the repression of HRas activation at the synapse established with sAg-loaded Raji cells, reinforcing the view that CD6 is fundamentally inhibitory.

Major advantages of FRET microscopy include the continuous monitoring of the enzymatic activity over time and, in the case of T-cell activation promoted by APCs, the ability to track the sensors and determine the site of activation. Little information is available regarding the spatio-temporal regulation of the different Ras members in T cells or T cell lines, except that KRas2B is expressed predominantly on the plasma membrane (PM), whereas NRas and HRas traffic between the PM and Golgi. Ras sub-cellular localization is mostly determined by the membrane-targeting domain (MTD, last 10–15 aa of Ras), which contains part of the HVR and the C-terminal CAAX box. Prenylation of the CAAX cysteine, followed by AAX tripeptide proteolysis and cysteine carboxymethylation enables Ras to loosely associate with membranes and also determines its addressing to the endoplasmic reticulum and Golgi [35, 75, 76]. Transport to the PM, however, requires a further posttranslational modification, the palmitoylation of yet other cysteine residue(s) within the MTD [35, 77]. HRas contains two cysteines that can be modified by palmitic acid, NRas and KRas2A contain one, and KRas2B has none [78]. Instead, a polybasic domain of six lysine residues in KRas2B can substitute for the palmitoylated cysteines as an alternative PM-targeting signal. Palmitoylation is reversible, and given that Ras acyltransferases are located in the Golgi while deacylating enzymes co-fractionate with the PM, Ras proteins can continually cycle between the different endomembrane compartments, except for KRas2B that resides mainly at the PM [79]. The FRET sensor for HRas that we developed, MRS2, was therefore modified to specifically replace the C-terminal sequence of KRas2B by that of HRas and thus faithfully report on the spatiotemporal mode of activation of the endogenous HRas.

Although it is undisputed that Ras responds to TCR triggering, there is controversy on whether HRas is activated on the Golgi apparatus [36, 39] or predominantly at the PM [37, 38]. In this study we have only observed the presence and activation of the MRS2 sensor at the PM during the formation of the T cell:APC synapse and never in the Golgi apparatus, supporting the view that the PM is the predominant site of Ras activation in Jurkat cells. However, caution should be taken to establish definitive conclusions, given that the mentioned studies used different methodologies and, to our knowledge, FRET-based live imaging of Ras has not been previously performed in T cells or T cell lines to be able to draw comparisons. Having maintained the same architecture of the extensively studied and validated Raichu FRET probes [58], in MRS2 we split the HRas sequence between the N- and the C-terminal ends within the HVR linker domain that connects the conserved N-terminal region and the MTD. It is therefore possible that the N-terminal 7 aa of the HVR that were separated from the MTD may have some additional roles, such as the interaction of Ras with lipid rafts, as previously suggested [80]. Although the topic of lipid rafts is also still very controversial regarding not only the biological function of these membrane microdomains but even their existence [81, 82], a detailed analysis of interaction of the sub-components of the MRS2 biosensor or variant probes with lipid rafts should deserve future consideration and help to unravel without any ambiguities the precise subcellular location of Ras activation in T lymphocytes.

One other limitation that FRET-based Ras/Raf-1 unimolecular biosensors may have is the permanent proximity imposed on the RBD to its Ras binding site via the linker, possibly artificially prolonging the measured Ras activation kinetics. The dislocation of FP modules may not be synchronous with the physiological Ras-RBD dissociation because the structure of the biosensor in an activated state may hinder the access of regulatory proteins (reviewed in [83]). In fact, using bimolecular or single-molecule FRET or biochemical methods in a number of different model systems, it has been shown that the activity of Ras peaks well before 5 min of stimulation, and decaying subsequently [84–86]. This contrasts with the sustained activation of HRas that we observe (at least 30 min after initial sAg recognition) using the unimolecular MRS2 biosensor. However, there are plenty of examples in the literature documenting that in T cell activation models the activity of Ras is sustained for long periods [87, 88], even exceeding 3 h after CD3 + CD28 mAb stimulation, which is fully in line with our observations. Moreover, in contrast with mAb activation triggered at the start of the conventional assays, our cellular system allows for T cell stimulation to be continuous as long as the T cell:APC pairs are engaged. Thus, although not excluding a minor contribution of the biosensor design to a prolonged recorded Ras activation, it is fully plausible that the MRS2 probe faithfully reproduces the behavior of Ras during physiological T cell activation.

The finding that RasGAP interacts with CD6 implied that the regulation of Ras could be a key part of the inhibitory function of CD6. Several other GAPs for Ras have been described in T cells [89] and, besides Raf-1, active Ras binds to a number of other effectors, including the lipid kinase PI3K, and RalGDS, a GEF for Ral GTPases [90, 91], each of these enzymes possibly leading to the activation of a variety of transcription factors. However, only RasGAP is equipped with an SH2 domain capable of binding to phosphorylated tyrosine residues of inhibitory receptors, acting immediately downstream of the TCR to restrain T-cell activation [23, 27]. As the absence of the cytosolic tail of CD6 seems to override the repression caused by the intact CD6 molecule in all steps of the canonical Ras-initiated MAPK signaling cascade, it seems more likely than ever that CD6 is an active repressor of signal transduction initiated by TCR triggering.

## Supplementary Information

**Additional file 1: Figure S1.** Gating strategy to detect CD69^+^ or CD25^+^ cells following sAg-mediated activation. E6.1-CD6WT and E6.1-CD6Dcyt cells were cultured for 24 h in the presence of unloaded or sAg-loaded Raji cells, and expression of CD69 and CD25 was assessed by flow cytometry. (a) The gate was defined based on unstained E6.1 cells, and positivity is represented in the right half of dot plots of FSC *vs.* marker expression. (b) Representative dot plots for each condition analyzing CD69 (left panels) or CD25 (right panels) expression. FSC: Forward scatter. **Figure S2.** Activity of the HRas biosensor MRS2 in unstimulated and activated E6.1 cells. (a) MRS2- and MRS2-S17N-expressing E6.1 single cells were filmed for 5 min at 37 ºC (1 min frames). At the 5 min mark, medium was added and cells were filmed for another 30 min. FRET/Clover ratios were assessed for each time-point. (b) Biosensor-expressing cells were initially filmed for 5 min alone. Then, unloaded or sAg-loaded Raji cells were added and allowed to form conjugates with the E6.1 cells for 30 min. FRET/Clover ratios were calculated for cells expressing MRS2 that interacted with Raji or Raji + sAg, and the same was performed for E6.1-MRS2-S17N:Raji pairs. (c) E6.1-CD6WT or E6.1-CD6∆cyt cells expressing either MRS2 or MRS2-S17N were filmed during conjugate formation with sAg-primed Raji cells. Biosensor-expressing E6.1 cells were filmed alone for 5 min, and for 30 min after sAg-Raji cells were added to the preparation. FRET/Clover ratios were calculated for each of the four experimental conditions. (a-c) Dots and lines represent mean ± SD of 5 independent experiments, with 10 individual cells (in a) or 9–10 cell pairs (in b and c) assessed for each condition in each experiment.

**Additional file 2: Movie S1.** Stability of the HRas FRET biosensor MRS2 in the absence of cell stimulation. (a) The activity of MRS2 was assessed using single cell live FRET microscopy. E6.1 cells expressing MRS2 were plated on poly-L-lysine-coated coverslips at 37 ºC and filmed for 5 min (1 min frames). At that point, medium was added and filming was resumed for another 30 min. FRET/Clover ratio is presented in a thermal scale (bottom right, range from 0.3 to 0.8). Images (two examples) are representative of 50 analyzed cells from 5 independent experiments. Full data are shown in Fig. S2a. (b) Activity of the inactive HRas biosensor MRS2-S17N, measured in the same conditions as in (a).

**Additional file 3: Movie S2.** MRS2 responsiveness to cell triggering. MRS2- or MRS2-S17N-expressing E6.1 cells were imaged using FRET live microscopy during conjugate formation with Raji cells. E6.1 cells were initially imaged alone and, after 5 min, filming was paused and Raji cells (unloaded or pre-loaded with sAg) were added to the preparation. Filming was resumed for an additional 30 min. FRET/Clover ratios are presented in a thermal scale (from 0.3 to 0.8). Two pairs of cells are shown for each condition, representative of 48–50 analyses for each E6.1:Raji combination, from 5 independent experiments. Full data are shown in Fig. S2b.

**Additional file 4: Movie S3.** CD6 downmodulates HRas activation. E6.1 cells expressing MRS2 or MRS2-S17N were further transduced with CD6WT or CD6∆cyt. Cells were imaged using live FRET microscopy as they formed synaptic conjugates with sAg-loaded Raji cells. E6.1 cells were initially imaged alone for 5 min, at which point imaging was paused and sAg-loaded Raji cells were added to the preparation. Filming was resumed for 30 additional min as synaptic contacts established. FRET/Clover ratios are presented in a thermal scale (from 0.3 to 0.8). Two pairs of cells are shown for each condition, representative of 48–50 analyses for each E6.1:Raji combination, from 5 independent experiments. Full data are shown in Fig. S2c.

## Data Availability

Expression vectors and established cell lines are available from the corresponding author upon reasonable request.
