# Supplementary material for: CD6-mediated inhibition of T cell activation via modulation of Ras
Source: Cell Commun Signal. 2022 Nov 21;20:184. doi: 10.1186/s12964-022-00998-x (PMC9682754; doi:10.1186/s12964-022-00998-x)
Supplement: Supplementary file 2 — Additional file 1: Figure S1. Gating strategy to detect CD69+ or CD25+ cells following sAg-mediated activation. E6.1-CD6WT and E6.1-CD6Dcyt cells were cultured for 24 h in the presence of unloaded or sAg-loaded Raji cells, and expression of CD69 and CD25 was assessed by flow cytometry. (a) The gate was defined based on unstained E6.1 cells, and positivity is represented in the right half of dot plots of FSC vs. marker expression. (b) Representative dot plots for each condition analyzing CD69 (left panels) or CD25 (right panels) expression. FSC: Forward scatter. Figure S2. Activity of the HRas biosensor MRS2 in unstimulated and activated E6.1 cells. (a) MRS2- and MRS2-S17N-expressing E6.1 single cells were filmed for 5 min at 37 ºC (1 min frames). At the 5 min mark, medium was added and cells were filmed for another 30 min. FRET/Clover ratios were assessed for each time-point. (b) Biosensor-expressing cells were initially filmed for 5 min alone. Then, unloaded or sAg-loaded Raji cells were added and allowed to form conjugates with the E6.1 cells for 30 min. FRET/Clover ratios were calculated for cells expressing MRS2 that interacted with Raji or Raji + sAg, and the same was performed for E6.1-MRS2-S17N:Raji pairs. (c) E6.1-CD6WT or E6.1-CD6∆cyt cells expressing either MRS2 or MRS2-S17N were filmed during conjugate formation with sAg-primed Raji cells. Biosensor-expressing E6.1 cells were filmed alone for 5 min, and for 30 min after sAg-Raji cells were added to the preparation. FRET/Clover ratios were calculated for each of the four experimental conditions. (a-c) Dots and lines represent mean ± SD of 5 independent experiments, with 10 individual cells (in a) or 9–10 cell pairs (in b and c) assessed for each condition in each experiment. [file 12964_2022_998_MOESM2_ESM.pdf]

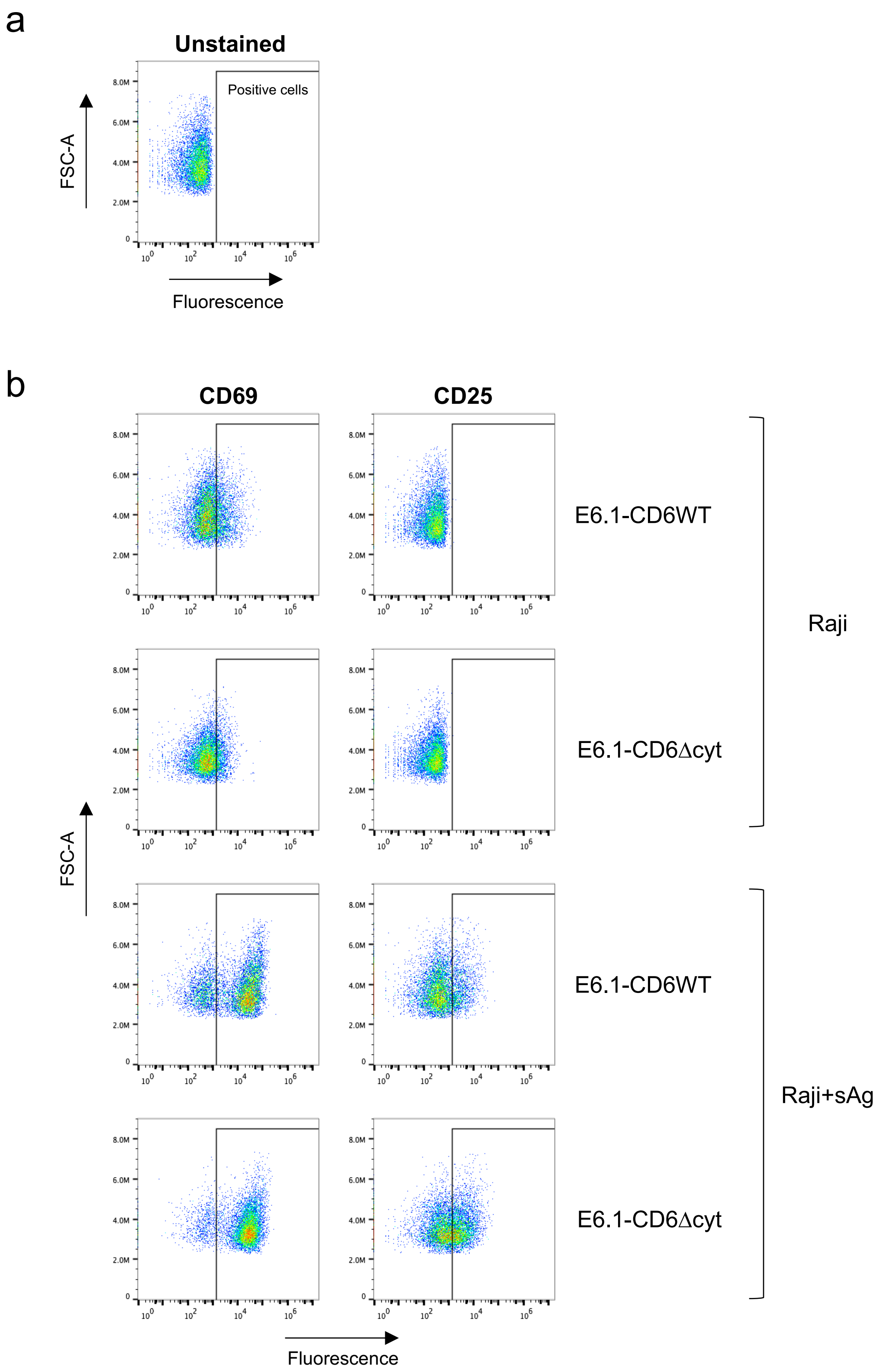

Figure S1. Gating strategy to detect CD69^{+} or CD25^{+} cells following sAg-mediated activation. E6.1-CD6WT and E6.1-CD6Δcyt cells were cultured for 24 h in the presence of unloaded or sAg-loaded Raji cells, and expression of CD69 and CD25 was assessed by flow cytometry. (a) The gate was defined based on unstained E6.1 cells, and positivity is represented in the right half of dot plots of FSC *vs.* marker expression. (b) Representative dot plots for each condition analyzing CD69 (left panels) or CD25 (right panels) expression. FSC: Forward scatter.

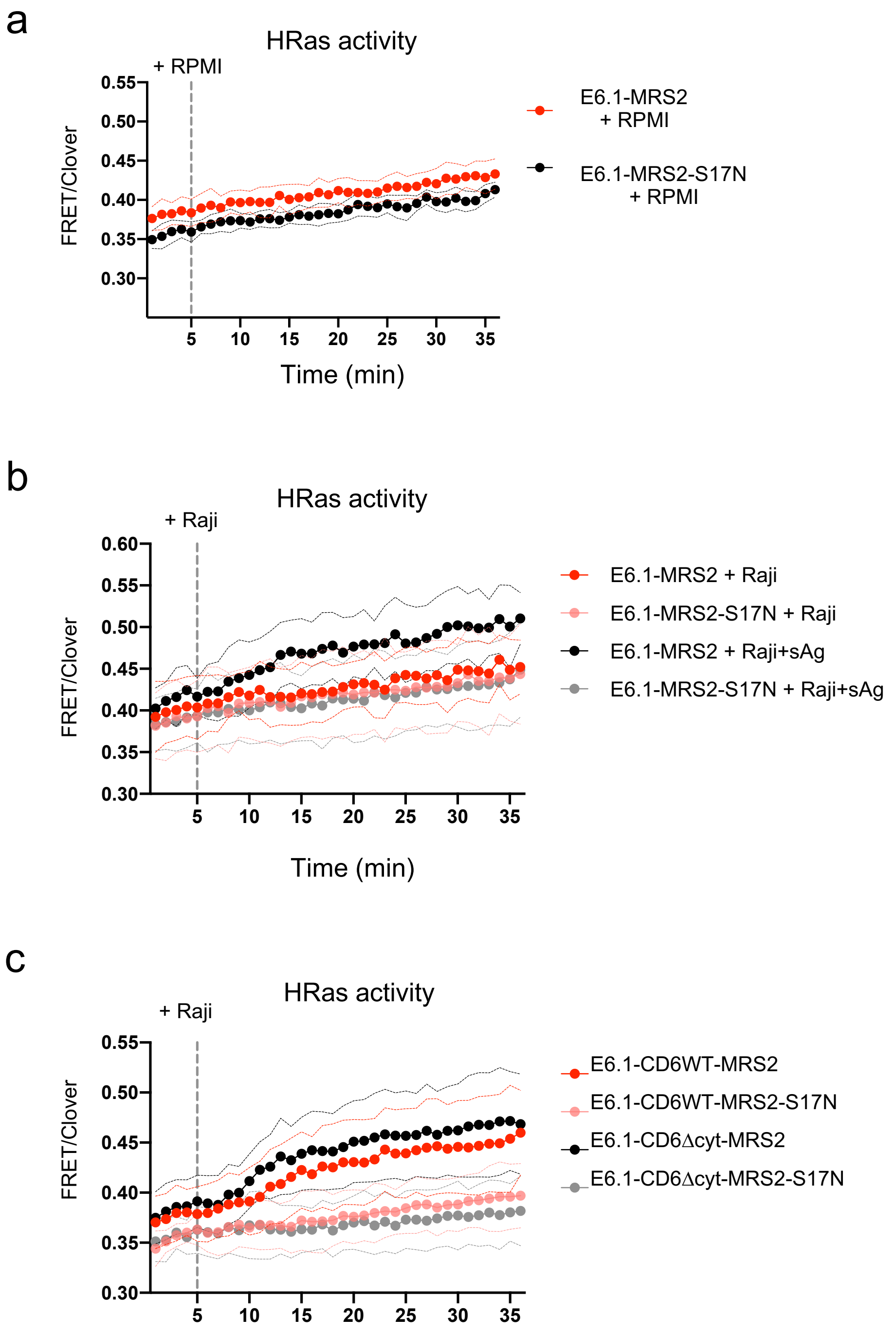

Figure S2. Activity of the HRas biosensor MRS2 in unstimulated and activated E6.1 cells. (a) MRS2- and MRS2-S17N-expressing E6.1 single cells were filmed for 5 min at 37 ºC (1 min frames). At the 5 min mark, medium was added and cells were filmed for another 30 min. FRET/Clover ratios were assessed for each time-point. (b) Biosensor-expressing cells were initially filmed for 5 min alone. Then, unloaded or sAg-loaded Raji cells were added and allowed to form conjugates with the E6.1 cells for 30 min. FRET/Clover ratios were calculated for cells expressing MRS2 that interacted with Raji or Raji+sAg, and the same was performed for E6.1-MRS2-S17N:Raji pairs. (c) E6.1-CD6WT or E6.1-CD6Δcyt cells expressing either MRS2 or MRS2-S17N were filmed during conjugate formation with sAg-primed Raji cells. Biosensor-expressing E6.1 cells were filmed alone for 5 min, and for 30 min after sAg-Raji cells were added to the preparation. FRET/Clover ratios were calculated for each of the four experimental conditions. (a-c) Dots and lines represent mean ± SD of 5 independent experiments, with 10 individual cells (in a) or 9-10 cell pairs (in b and c) assessed for each condition in each experiment.
